# Factors Associated with Oral Cancerous and Precancerous Lesions in an Underserved Community: A Cross-Sectional Study

**DOI:** 10.3390/ijerph19031297

**Published:** 2022-01-24

**Authors:** Parvaneh Badri, Hollis Lai, Seema Ganatra, Vickie Baracos, Maryam Amin

**Affiliations:** 1 Faculty of Medicine and Dentistry, School of Dentistry, University of Alberta, Edmonton, AB T6G 1C9, Canada; badri@ualberta.ca (P.B.); hollis1@ualberta.ca (H.L.); sganatra@ualberta.ca (S.G.); 2 Department of Oncology, Cross Cancer Institute, Faculty of Medicine and Dentistry, University of Alberta, Edmonton, AB T6G 1Z2, Canada; vbaracos@ualberta.ca

**Keywords:** oral cancer screening, high-risk population, health inequality

## Abstract

Street-involved people with limited access to regular healthcare have an increased risk of developing oral cancer and a lower survival rate. The objective of this study was to measure the prevalence of oral cancerous/precancerous lesions and determine their associated risk factors in a high-risk, underserved population. In this cross-sectional study, English-speaking adults aged 18 years and older living in a marginalized community in Edmonton were recruited from four non-profit charitable organizations. Data were collected through visual oral examinations and a questionnaire. Descriptive statistics, chi-squared tests, and logistic regressions were applied. In total, 322 participants with a mean (SD) age of 49.3 (13.5) years completed the study. Among them, 71.1% were male, 48.1% were aboriginal, and 88.2% were single. The prevalence of oral cancerous lesions was 2.4%, which was higher than the recorded prevalence in Canada (0.014–1.42: 10,000) and in Alberta (0.011–1.13: 10,000). The clinical examinations indicated that 176 (54.7%) participants had clinical inflammatory changes in their oral mucosa. There was a significant association between clinical inflammatory oral lesions and oral cancerous/precancerous lesions (*p* < 0.001). Simple logistic regression showed that the risk of the presence of oral cancerous/precancerous lesions was two times higher in participants living in a shelter or on the street than in those living alone (OR = 2.06; 95% CI: 1.15–3.82; *p*-value: 0.021). In the multiple logistic regression analysis, the risk of oral cancerous/precancerous lesions was 1.68 times higher in participants living in a shelter or on the street vs. living alone after accounting for multiple predictors (OR = 1.67; 95% CI: 1.19–2.37; *p*-value: 0.022). The results demonstrated a high prevalence of cancerous/precancerous lesions among the study participants, which was significantly associated with clinical inflammatory oral lesions. The evidence supports the need for developing oral cancer screening and oral health promotion strategies in underserved communities.

## 1. Introduction

Oral health is a general reflection of overall health. Oral cancer/precancerous lesions are serious health conditions that could tremendously affect a patient’s overall health and quality of life, particularly if not detected and treated. Over 90% of oral cancers are diagnosed as oral squamous cell carcinoma (OSCC), which has a high mortality rate and comprises 30% of all head and neck malignancies [1,2,3]. Head and neck cancers comprise 4% of cancer incidences in the United States and Canada [4]. Most oral precancerous and early cancerous lesions are symptomless, resulting in the pursuit of medical attention at advanced stages and leading to a poor prognosis and low survival rate [3]. This is an unfortunate outcome considering that high-risk precancerous lesions are easily detected by a visual examination of the mouth. Leukoplakia (white changes), erythroplakia (red changes), and erythroleukoplakia (a combination of white and red changes) represent the most common oral precancerous lesions, defined as clinical white/red patches that cannot be rubbed off and clinically cannot be characterized for any diseases [5].

Cancer etiology, risk factors, and interventions are not one-size-fits-all across different populations [6]. According to the World Health Organization, oral cancer varies in distribution from region to region. Two-thirds of the global incidence of oral cancer occurs in low- and middle-income regions such as South Asia [2]. Recent evidence indicates that health inequality in Canada persists and that unequal distribution of wealth along with social determinants of health resources lead to inequity in health outcomes in lower socioeconomic classes [7,8]. The groups known to be at higher risk for developing chronic diseases such as oral malignancies have had worse survival rates and significantly reduced quality of life [9,10]. Researchers in British Columbia and Ontario investigated how oral malignancy incidence varied according to a neighborhood’s socioeconomic status (SES), including a magnitude of inequalities [11,12]. Their findings support community-based interventions to address barriers to access to care, including the delivery of health education and promotion programs among the most SES-deprived [11].

Similar to SES deprivation that could be associated with chronic diseases and cancers, chronic inflammation, such as inflammatory changes of oral mucosa at high-risk sites (e.g., lateral border of the tongue, floor of the mouth, and oropharynx, as well as periodontal diseases), can contribute significantly to the process of developing oral cancer [13]. While inflammation is a biological response of body tissues to harmful stimuli, pathogens, damaged cells, or trauma [14], oral inflammation is any inflammatory process affecting the mucous membrane of the mouth and lips with/without ulceration [15].

While previous studies have shown the associations between SES deprivation and the incidence of potential oral malignancies [4,11,16], different geographic locations with distinct sociodemographic, cultural, lifestyle, and access-to-care diversities may show a different pattern of SES deprivation vulnerability towards oral cancer. Therefore, the objectives of the present study were to measure the prevalence of oral cancerous/precancerous lesions within a high-risk underserved population and to investigate the associated risk factors for these lesions in this population.

## 2. Materials and Methods

A cross-sectional study was conducted between 2017 and 2020 in a marginalized high-risk community in Edmonton, Canada. Ethics approval was obtained from the University of Alberta Research Ethics Board (Pro00060953).

### 2.1. Setting and Sampling

Participants were English-speaking adults who were older than 18 years and living in the Boyle–McCauley Street community. We conducted our study mainly in the Boyle community because it is a well-known and officially recognized community that has been designated by public health and other authorities in Edmonton as having a population that experiences poverty, homelessness, mental health, addiction, and social isolation. The Boyle–McCauley Street area is one of the most high-risk and underserved communities in Edmonton. It is located in central Edmonton, just east of the city’s downtown core [17]. The participants were recruited from four major non-profit charitable centers: Boyle McCauley Health Centre Dental Clinic [18]; George Spady Society Shelter, Detox, and Supervised Consumption Service [19]; Operation Friendship Seniors Society [20]; and Bissell Centre West [21]. These centers provide relevant and accessible primary healthcare and wellbeing support to in-need community members. However, for unknown reasons, a higher proportion of men than women access the above centers. Executive directors of these organizations facilitated the recruitments through their centers and connections with the community. All participants received an honorarium at the completion of the study. A sample size of 360 was estimated based on the population residing in the community (6240 adult, ≥18 years), with a 95% confidence interval and a 5% error margin.

### 2.2. Data Collection

Once consent was obtained, data were collected through clinical oral examinations and a questionnaire consisting of demographics, behavioral risk factors, oral health perceptions and behaviors, medical history, and healthcare utilization. The questionnaire was adopted from the American Academy of Oral Medicine Clinician’s Guideline [5]. The clinical examinations were performed by two calibrated examiners. Two certified, experienced dental practitioners, using a mobile dental chair and LED lamp at the centers, independently performed examinations on thirty patients for the calibration of DMFT scores (k = 0.83) and oral lesions (k = 0.83), with agreement in 29 of the 30 cases reviewed (corresponding Cohen’s Kappa interpretation category of 0.80–1.00). Participants diagnosed with oral cancerous and precancerous lesions were referred to the Boyle McCauley Dental Centre, which is affiliated with the University of Alberta School of Dentistry, for further assessments. Clinical measures included (1) oral lesions and (2) DMFT exams. DMFT scores range from 0 to 28 and refer to the number of identified caries on 28 adult teeth, excluding wisdom teeth (8 s). The score 0 represents no caries, and the score 28 represents that those 28 teeth were diagnosed with caries. We followed standard biosafety and infection control protocols for participants and the examiners to prevent infections during our clinical examinations, guided by the Canadian biosafety standard [22]. Sterilized mirrors, probes, and gauze were used for the clinical examinations. We defined oral inflammation as any changes in the oral mucosa presenting as red, white, or pink, traumatic conditions in various dimensions and locations with/without pain, and function limitations clinically detected in oral cavity. Histopathological examinations as a definitive diagnosis of inflammation were not conducted in this study.

### 2.3. Statistical Analysis

Our outcome variable was the presence of oral cancerous/precancerous lesions, dichotomously categorized as “1” for presence and “0” for absence of the lesion. Student’s *t*-test was used to examine the continuous variables differences in patients with and free of lesions. To determine whether a proportion between the outcome variable and all categorical variables exists, the chi-squared test with Phi and Cramer’s V option were conducted. Univariate analyses determined the associations between sociodemographic characteristics (including age, educational level, and living status) and the presence of lesions. Adjusted logistic regression was conducted to examine the risk of oral cancerous/precancerous lesions. The coefficient of determination (r2) of the regression model was calculated. In addition, a test of normality was performed, and the data are presented in OR, 95% CI from. An alpha level of 0.05 was used to determine statistical significance. All analysis was conducted using the Statistical Package for Social Sciences (SPSS for Windows, version 24.0; SPSS Inc., Chicago, IL, USA).

## 3. Results

A total of 322 participants completed the study. All the volunteer candidates from the four major non-profit charitable centers agreed to participate in the study. We had no non-responsive participants. The participants were 18–97 years old, with a mean (SD) age of 49.3 (13.5). Of all, 71.1% of participants were male, 48.1% were aboriginal, and 88.2% were never married/divorced/separated. The participants’ sociodemographic characteristics are presented in Table 1.

For substance use, 68.6% of the participants used tobacco, 55.9% recreational drugs, and 52.8% alcohol (Table 2).

Participants had a better perception of their general health compared to their oral health. While 63% perceived their oral health status as fair to poor, only 38.8% had a fair-to-poor perception of their general health (Table 3).

Furthermore, 62.4% of the participants had no history of cancer screening, and 33.2% claimed they had no access to care when needed. The descriptive analysis identified a higher point estimate for the categories of those aged 45–65 years, an education level lower than Grade 10, smoking, tobacco exposure higher than 20 years, recreational drugs usages, alcohol usage, fair-to-poor oral health perceptions, and not having heard about oral cancer among the group with oral cancerous/precancerous lesions compared to their counterpart cohort (Table 4).

The clinical assessments indicated that 60 (18.6%) participants presented with oral cancerous/precancerous lesions; of them, five cases (1.5%) were diagnosed with squamous cell carcinoma (Table 5).

It was not technically feasible to perform a histopathological examination as a definitive diagnosis for each detected lesion. Participants with cancerous/precancerous lesions were referred for follow-up by a dental clinic, and exceptional biopsies were performed for highly suspicious advanced lesions detected in five participants to avoid further delay in their management.

Clinical inflammatory oral mucosa changes were detected in 176 (54.7%) cases at various sites, including ventral/dorsal/border of the tongue, buccal mucosa, floor of the mouth, retromolar pads, tonsils, and oral gingiva. Almost 62% of participants had a high categorical level of DMFT (score of 9 or more), ranging from 0 to 28 for dental caries, with a mean (SD) of 13.4 (7.20).

Student’s *t*-test was not significant for continuous variables. In the univariate analyses, the clinical inflammatory oral mucosal changes were related to oral cancerous/precancerous lesions in participants (K2, DF, *p*-value, and Phi = 48.24, 1, 0.001, and 0.38, respectively; 0.29–0.47). To analyze the association between oral cancerous/precancerous lesions and participants’ living status, simple logistic regression was conducted. The risk of presence of oral cancerous/precancerous lesions was two times higher in participants living in a shelter/on the street than those living alone (OR = 2.06; 95% CI: 1.15–3.82; *p*-value: 0.021). A multiple logistic regression analysis was then conducted to examine the risk of the presence of oral cancerous/precancerous lesions with living status and clinical inflammatory oral mucosal changes. The risk of oral cancerous/precancerous lesions was 1.68 times higher in participants living in a shelter/on the street vs. living alone after accounting for the multiple predictors (OR = 1.67; 95% CI: 1.19–2.37; *p*-value: 0.022) (Table 6 and Table 7). The coefficient of determination (r2) of the regression model was calculated at 0.310.

## 4. Discussion

This study confirmed our hypothesis that the prevalence of oral cancerous/precancerous lesions in the Boyle–McCauley Street community was higher than the reported prevalence in Canada (0.014–1.42%:10,000) and in Alberta (0.011–1.13%:10,000). The prevalence of 1.5% for cancerous lesions in our study is anticipated to increase to 2.4%, because 5% of the 55 cases of precancerous lesions are also expected to transform into a malignancy [23,24]. The point prevalence per 10,000 people was calculated for Canada and Alberta based on the total count of new cases of oral cancer in 2020 [25,26] divided by the reported quarterly population count for Canada [27] and Alberta [28] and multiplied by 10,000. From the 322 participants in this study, we identified 55 (17.1%) cases of oral precancerous lesions and five cases of confirmed cancer (COVID-19 outbreak interfered with the histopathological biopsy for most cases with oral precancerous lesions that this study identified). This finding shows a 1.6% oral precancerous lesion and 0.7% cancerous lesion increase rate compared to the findings of 15.5% oral precancerous lesions and 1.7% confirmed cancer in a similar study conducted in Vancouver, BC [29], whereas Lim et al., in their study conducted on general dental practices in England, reported 4.2% oral precancerous lesions among 2265 patients [30].

We found a positive association between the “living in shelter/street” status and oral precancerous lesions. This result is consistent with previous studies reporting the influence of environmental quality and an individual’s living status on cancer incidence [6,31]. A number of physical, infectious, and mental health issues are known to be associated with homelessness and living in shelters [32,33,34]. Additionally, despite living with others (mostly strangers), individuals living in shelters experience a significant psychological loneliness caused by a lack of desired support from family or friends. This demands special attention, as the evidence indicates that the experience of loneliness of homeless individuals is significantly different compared to the general population, and it is strongly associated with an increased risk of cancer incidence and mortality [35,36,37,38,39]. Feeling lonely and isolated is also associated with health risk behaviors such as low intake of fruit or vegetables, daily smoking, high-risk alcohol intake, and physical inactivity, as well as their co-occurrence [35,36,38,39].

In 2019, 53,000 North Americans were diagnosed with oral cancer, resulting in 9750 deaths [40]. The 2019 Canadian Cancer Statistics reported 5300 Canadians who were diagnosed with oral cancer (3700 men and 1600 women), of which 1480 died (1050 men and 430 women) [41]. Furthermore, Alberta is positioned fourth, after Ontario, Quebec and British Columbia, in terms of oral cancer incidence and related death prevalence among Canada’s provinces and territories [41].

Johnson et al. identified an increased rate of head and neck cancer (HNC) in Canada and the United States among individuals of lower socioeconomic status [4]. In their study, potential risk factors associated with HNC such as income level, education grade, annual dental visits, and immigration status were investigated. Individuals with lower median family income, adults with less than a Grade 8 education, and individuals who visited a dentist less than once a year had a significantly higher rate of HNC incidence. Hwang et al. [10] explored HCN incidence and socioeconomic status in Canada between 1992–2007. The study showed that the incidence rate was significantly higher in cases with lower income quintiles. The results of a study conducted in British Columbia also supported a higher rate of HCN incidence in individuals who were living in the most deprived geographic location, were unemployed, and had a lower level of education [16]. However, in a fully adjusted multivariate analysis, smoking was the only significant risk factor [16]. Similarly, tobacco smoking, alcohol consumption, lower household income, and lower education level were identified as the main risk factors significantly associated with head and neck squamous cell carcinoma in North Carolina [42]. By contrast, in the present study, we noted that important factors such as age (45–65 years), education level (lower than grade ten), smoking and tobacco exposure (longer than 20 years), recreational drug use, alcohol use, fair to poor oral health perceptions, and not having heard about oral cancer had a higher point estimate among the oral cancerous/precancerous lesions group but were not statistically significant. The reason might be our sample size, which was calculated to provide the optimum statistical power in a cross-sectional study. Most of the previous reports on the impact of SES on the incidence of oral cancer/head and neck cancers in underserved populations were designed retrospectively, using a large number of national and international administrative data across a consecutive number of years with stronger statistical power [4,10,11,12,16,43,44].

We identified a significant positive association between clinical inflammatory oral mucosal changes and oral cancerous/precancerous lesions. The role of chronic inflammation (i.e., a pathological response of the body to nocuous stimuli) in carcinogenesis has been proven since 1963 by many studies [45]. In addition, there is consensus in the literature that tobacco and alcohol users with a poor diet and living in deprived communities similar to Boyle–McCauley Street are at higher risk of developing oral inflammatory diseases and precancerous lesions with a higher risk of progression to malignancy. Nonetheless, there is an early and cost-effective opportunity to detect cancerous or precancerous changes of the oral cavity visually. The high rate of oral cancer/precancerous lesions and their significant association with oral mucosal inflammation in this study urge implementation of an effective preventive health strategy. While, to the best of our knowledge, there is no provincial program for oral cancer screening in Alberta [46], our accumulated evidence supports the benefits of periodic, cost-effective, opportunistic/targeted oral cancer screening for high-risk populations residing in underserved communities.

**Study limitations:** It is worth noting that we experienced significant challenges during our data collection. This study does not claim to establish any cause-and-effect relationships between the variables used in the research. We tested the associations between dependent (oral cancerous/precancerous lesions) and independent variables (demographic characteristics, behavioral risk factors, oral health perceptions and behaviors, medical history, and healthcare utilization. There were also technical difficulties that interfered with data collection at community centers and shelters. For instance, it was not technically feasible to perform a histopathological examination as a definitive diagnosis for each detected lesion, and the inclusion of maxillofacial radiographic bony lesions was unfortunately not feasible in this study. In addition, our target population was hard to reach. It took us a significant amount of time to build trust among all parties, including the research team, centers’ administrations, and eligible participants who distrust researchers due to their unpleasant past experiences. Furthermore, the research team faced difficulties in acquiring answers to some of the sensitive questions such as ethnic background and annual income. However, we were able to build a strong and positive connection with the community partners and participants that allowed us to collect necessary information from a hard-to-reach population.

## 5. Conclusions

The high prevalence of cancerous/precancerous lesions found in an underserved community in our study supports the findings that deprived socioeconomic communities are at high risk of being victimized by inequality in health outcomes. While we only found oral mucosal inflammatory changes and living status as predictors of cancerous/precancerous lesions, exposure to tobacco, alcohol, and recreational drugs may also attribute to the development of precancerous lesions. These facts urge the development of an opportunistic oral cancer screening health promotion strategy using the available infrastructure and potentials in high-risk communities. Additionally, the comprehensive multi-level collected data in this study, including demographics, risk factors, and general health and oral health in a high-risk underserved population, could provide a baseline source of reference for future research in this field. Effective public health awareness campaigns about oral cancer and its associated risk factors, particularly among at-risk populations, are recommended.

## Figures and Tables

**Table 1:** Demographic characteristics (*N* = 322).

Variable	Category	*N* (%)
Sex	Female	93 (28.9)
	Male	299 (71.1)
Age	Mean	49.3 13.5
	Standard deviation	
Ethnicity	White/Caucasian	115 (35.7)
	Other ethnic background	155 (13.7)
	Aboriginal	44 (48.1)
	Declined to answer	8 (2.5)
Education level (yr.)	>12	84 (26.1)
	10–12	114 (35.4)
	<10	124 (38.5)
Marital status	Married/common law	36 (11.2)
	Divorced/separated	100 (31.1)
	Never married	184 (57.1)
	Declined to answer	2 (0.6)
Living status	With family	51 (15.8)
	Alone (house/apartment)	147 (46.7)
	In shelter/street	121 (37.6)
	Declined to answer	3 (0.9)
Employment status	Working	35 (10.9)
	Not working	287 (89.1)
Annual income ($ CAD)	>$12,000	77 (23.9)
	<6000–12,000	180 955.9)
	Declined to answer	65 (20.2)
Financial aids (governmental and non-governmental financial assistance for low-income people)	Yes	225 (69.9)
	No	81 (25.2)
	Declined to answer	61 (5.0)
Living at Boyle Street	≤3 Months	62 (19.3)
	3–6 Months	40 (12.4)
	6–12 Months	27 (8.4)
	>12 Months	193 (59.9)

Note: yr. = Year; $ CAD = Canadian Dollar

**Table 2:** Risk factors (tobacco/recreational drug/alcohol use) (*N* = 322).

Variable	Category	*N* (%)
Smoking tobacco	Yes	221 (68.6)
	No	101 (31.4)
Tobacco exposure in years	<20	42 (13.0)
	≥20	179 (55.6)
	Non-users	101 (31.3)
Starting age tobacco use	<15	119 (36.9)
	15–18	71 (22.0)
	>18	31 (9.6)
	Non-users	101 (31.3)
Quantity smoked per day	<20	146 (45.3)
	≥20	75 (23.2)
	Non-users	101 (31.3)
**Recreational drug use**	Yes	180 (55.9)
	No	142 (44.1)
Starting age recreational drug use	<20	120 (37.3)
	≥20	60 (18.6)
	Non-users	142 (44.1)
Frequency of recreational drug use	Occasional	8 (2.5)
	Often	93 (28.9)
	Every day	79 (24.5)
	Non-users	142 (44.1)
**Type of recreational drug**	Marijuana	54 (16.8)
	Crack/cocaine	14 (4.3)
	Crystal meth	24 (7.5)
	Methadone	3 (0.9)
	Mixed	85 (26.4)
	Non-users	142 (44.1)
**Alcohol consumption**	Yes	170 (52.8)
	No	152 (47.2)
**Start age alcohol consumption**	<15	76 (23.6)
	15–20	77 (23.9)
	>20	17 (5.2)
	Non-users	152 (47.2)
**Type of alcohol**	Beer	53 (16.4)
	Wine	7 (2.1)
	Liquor/shots	30 (9.3)
	Mixed	81 (25.1)
	Non-users	152 (47.2)
**Years of alcohol consumption**	<20	37 (11.4)
	≥20	133 (41.3)
	Non-users	152 (47.2)

**Table 3:** Oral health perceptions and behaviors (*N* = 322).

Variable	Category	*N* (%)
Oral health perception	Excellent	5 (1.6)
	Very good	22 (6.8)
	Good	64 (19.9)
	Fair	108 (33.5)
	Poor	99 (30.7)
	Declined to answer	24(7.5)
Recent dental visit	Never	9 (2.8)
	Within the past year	116 (36.0)
	Within 1–5 years	123 (38.2)
	>5 years	74 (23.0)
Uncomfortable to eat or drink in the past month	Never	140 (43.5)
	Once a week	44 (13.7)
	More than once	132 (41.0)
	Declined to answer	6 (1.8)
What bothers you most about your mouth/teeth	Nothing	71 (22.0)
	Eating	145 (45.0)
	Others (talking/appearance)	106 (32.9)
Main oral problem	Pain	134 (41.7)
	Others (sharp and missing teeth, bad breath, ill-fitting dentures)	114 (35.4)
	Declined to answer	74 (23.0)
History of head and neck cancer in family	Yes	30 (9.3)
	No	292 (90.7)
Heard about oral cancer	Yes	171 (53.1)
	No	151 (46.9)
Oral cancer screening in the past	Yes	33 (10.2)
	No	285 (88.5)
	Declined to answer	4 (1.2)

**Table 4:** Oral cancerous/precancerous lesions group vs. cancer-free group.

Factors	Category	With Cancer/Precancerous *N* = 60 *N* (%)		Unidentified Precancerous *N* = 262 *N* (%)	
Age	≤44	20 (33.3)		94 (35.9)	
	45–65	34 (56.7)		137 (52.3)	
	>65	6 (10)		31 (11.8)	
Age		Mean SD	50.43	Mean	49.03
			12.0	SD	13.5
Education level (yr.)	>12 10–12 <10	16 (26.7)		68 (26)	
		16 (26.7)		98 (37.4)	
		28 (46.7)		96 (36.6)	
Marital status	Married/common Law	8 (13.3)		28 (10.7)	
	Divorced/separated	20 (33.3)		80 (30.5)	
	Never married	32 (53.3)		152 (58.0)	
	Declined to answer	0 (0.0)		2 (0.7)	
Living status	With family	7 (11.7)		44 (17.0)	
	Alone (house or apartment)	21 (35.0)		126 (48.0)	
	Shelter/street	31 (51.7)		90 (34.3)	
	Declined to answer	1 (1.7)		2 (0.7)	
Annual income ($ CAD)	>$12,000 <$6000–12,000 Declined to answer	15 (25.0)		62 (23.7)	
		31 (51.6)		149 (56.8)	
		14 (23.3)		51 (19.5)	
Financial aid	Yes	41 (68.3)		184 (70.2)	
	No	15 (25.0)		66 (25.2)	
	Declined to answer	4 (6.7)		12 (4.6)	
Clinically inflammatory oral mucosal	No	3 (5.0)		143 (54.6)	
	Yes	57 (95.0)		119 (45.4)	
Smoke tobacco	No	18 (30.0)		83 (31.7)	
	Yes	42 (70.0)		179 (68.3)	
Tobacco exposure (yr.)	<20	5 (8.3)		37 (14.1)	
	≥20	37 (61.7)		142 (54.2)	
	Non-users	18 (30.0)		83 (31.7)	
Recreational drug use	No	26 (43.3)		116 (44.3)	
	Yes	34 (56.7)		146 (55.7)	
Alcohol usage	No	28 (46.7)		124 (47.3)	
	Yes	32 (53.3)		138 (52.70	
Oral health perception	Excellent	2 (3.3)		3 (1.1)	
	Very good	4 (6.7)		18 (6.9)	
	Good	8 (13.3)		56 (21.4)	
	Poor to fair	40 (66.7)		167 (63.7)	
	Declined to answer	6 (10.0)		18 (6.9)	
Heard about oral cancer	Yes	29 (48.3)		142 (54.2)	
	No	31 (51.7)		120 (45.8)	

Note: yr. = Year; $ CAD = Canadian Dollar

**Table 5:** Oral mucosal screening (participants: *N* = 322).

Variable	*N*	Total Participants *N* = 322 (%)	
Inflammatory oral mucosal changes	176	54.7	
Oral precancerous lesions	55	17.1	
Squamous cell carcinoma confirmed by biopsy	5	1.5	
Categories of precancerous lesions prior to biopsy	***N***	***N*** **= 322 (%)**	**Precancerous *N* = 60** **(%)**
Leukoplakia	40	12.4	66.6
Erythroplakia	1	0.3	1.7
Erythroleukoplakia	3	1.0	5.0
Lichen planus	1	0.3	1.7
Submucous fibrosis	1	0.3	1.7
Highly suspicious nonhealing ulcers	14	4.3	23.3
	60	18.6%	100

**Table 6:** Statistical analysis—oral cancerous/precancerous lesions associations.

Univariate Analysis					
Clinical inflammatory oral mucosa changes	^a^ K2 48.24	^b^ DF (1)	*p*-value 0.001	^c^ Phi 0.38	95% CI 0.29–0.47
**Logistic Regression Analysis**		Unadjusted			
Participant’s living status	OR 2.06	95% CI 1.15–3.82	*p*-value 0.021		
Participant’s living status		Adjusted			
	OR 1.67	95% CI 1.19–3.37	*p*-value 0.022		

^a^ K2 measures the difference of observed numbers with theoretically expected numbers with regards to degree of freedom in 2 × 2 table; ^b^ DF—degrees of freedom; ^c^ Phi—Phi Coefficient (measures the association strength between two variables in Chi-square test); underline—the value is under the underlined title.

**Table 7:** Statistical analysis—multiple regression.

Variables	OR	95% CI
**Sex**	1.54	0.77–3.04
**Age**	1.04	0.99–1.09
**Ethnicity**	2.03	0.29–4.01
**Education**	0.71	0.32–1.58
**Marital status**	0.96	0.61–1.51
**Housing**	1.81	0.47–6.8
**Employment**	0.74	0.27–2.00
**Financial aid**	0.50	0.64–3.28
**Annual income**	0.36	0.64–3.28
**Duration of living in Boyle–McCauley Street**	0.66	0.22–2.01

## Data Availability

The supporting generated data during the study can be found at the University of Alberta School of Dentistry, Edmonton, Alberta, Canada.
